# The Mechanical and Physical Properties of 3D-Printed Materials Composed of ABS-ZnO Nanocomposites and ABS-ZnO Microcomposites

**DOI:** 10.3390/mi11060615

**Published:** 2020-06-25

**Authors:** Nectarios Vidakis, Markos Petousis, Athena Maniadi, Emmanuel Koudoumas, George Kenanakis, Cosmin Romanitan, Oana Tutunaru, Mirela Suchea, John Kechagias

**Affiliations:** 1 Mechanical Engineering Department, Hellenic Mediterranean University, 71004 Heraklion, Greece; vidakis@hmu.gr; 2 Department of Materials Science and Technology, University of Crete, 70013 Heraklion Crete, Greece; maniadi@materials.uoc.gr; 3 Department of Electrical and Computer Engineering, Hellenic Mediterranean University, Estavromenos, 71004 Heraklion, Greece; koudoumas@hmu.gr (E.K.); mira.suchea@imt.ro (M.S.); 4 Institute of Electronic Structure and Laser, Foundation for Research and Technology-Hellas, 70013 Heraklion, Greece; gkenanak@iesl.forth.gr; 5 National Institute for Research and Development in Microtechnologies (IMT-Bucharest), 077190 Bucharest, Romania; cosmin.romanitan@imt.ro (C.R.); oana.tutunaru@imt.ro (O.T.); 6 General Department, University of Thessaly, 41500 Larissa, Greece; jkechag@uth.gr

**Keywords:** additive manufacturing, 3D printing, fused filament fabrication (FFF), acrylonitrile butadiene styrene (ABS), nanocomposites, tensile, flexural, strength

## Abstract

In order to expand the mechanical and physical capabilities of 3D-printed structures fabricated via commercially available 3D printers, nanocomposite and microcomposite filaments were produced via melt extrusion, 3D-printed and evaluated. The scope of this work is to fabricate physically and mechanically improved nanocomposites or microcomposites for direct commercial or industrial implementation while enriching the existing literature with the methodology applied. Zinc Oxide nanoparticles (ZnO nano) and Zinc Oxide micro-sized particles (ZnO micro) were dispersed, in various concentrations, in Acrylonitrile Butadiene Styrene (ABS) matrices and printable filament of ~1.75 mm was extruded. The composite filaments were employed in a commercial 3D printer for tensile and flexion specimens’ production, according to international standards. Results showed a 14% increase in the tensile strength at 5% wt. concentration in both nanocomposite and microcomposite materials, when compared to pure ABS specimens. Furthermore, a 15.3% increase in the flexural strength was found in 0.5% wt. for ABS/ZnO nano, while an increase of 17% was found on 5% wt. ABS/ZnO micro. Comparing the two composites, it was found that the ABS/ZnO microcomposite structures had higher overall mechanical strength over ABS/ZnO nanostructures.

## 1. Introduction

Fused filament fabrication (FFF) is a 3D printing process, in which a selected material is extruded layer by layer to create a larger 3D object [1,2]. While almost every base material—such as sugars [3], thermoplastics and thermoplastic composites [4], photopolymers [5], glass [6], metals [7], metal oxides [8], and ceramic based composites [9]—can be utilized in 3D printing in order to print complex 3D structures, a strong demand exists for novel materials specifically designed for 3D printing applications, that can improve the mechanical and physical properties of 3D printed structures, offering greater functionality.

The mechanical properties of 3D-printed ABS parts [10,11,12,13] are changing, depending on the 3D printing process parameters selected among other factors, in addition to the fact that Fused filament fabrication (FFF) introduces anisotropy to the final parts. Regarding composite and nanocomposite structures, 3D printed parts have natural striations resulting from the 3D printing resolution. This is because the 3D printing process affects the homogeneity of the composites and thus alters the properties of the final material [4].

Nanocomposite and microcomposite filaments for 3D printing, are nowadays in high demand due to the nanoscale and microscale interactions. These interactions can improve the mechanical stability and durability of the resulting materials [14,15,16,17]. Studies have also shown that fillers in nano scale, can be more uniformly dispersed in polymeric matrices, leading to higher mechanical toughness when compared to composites filled with filler particles in micro scale [18,19].

Regarding nano and micro fillers used in composite filaments, there are several literature reports regarding nano materials such as Titanium Oxide (TiO) [4], Multiwalled Carbon Nano Tubes (MWCNTs) [20,21] and Graphene Nano Platelets (GnPs) [20,22]. The aim of these studies was to improve the final properties of the composites.

This work regards ABS composites incorporating two inorganic fillers: Zinc Oxide nano particles (ZnO nano) and Zinc Oxide micro particles (ZnO micro)—both of high interest, due to the properties of Zinc Oxide, which are among others its chemical stability, its semi conductivity and its overall high mechanical toughness [23,24,25,26]. ABS was selected as the polymer matrix because it is widely used in several industries, and especially for 3D printing due to its desirable physical and mechanical properties [27]. Several studies have been published [28,29,30,31,32,33,34] on the mechanical properties of the ABS material and its composites under different manufacturing scenarios, but none has focused so far on the mechanical and physical properties of 3D printed specimens built with ABS as the polymer matrix and ZnO as the filler. Furthermore, no literature has studied yet both nanocomposites and microcomposites to determine the effect of the filler scale on the composite properties.

Therefore, ABS/ZnO nano and micro-composites are extremely attractive compounds to be used in a variety of nanotechnology applications. Some of these applications include biomedicine, energy, sensors, and optics [25]. Regarding melt extrusion and 3D printing, both have the advantage to be completely solvent-free methods for fabricating homogeneous ABS/ZnO nanocomposites and microcomposites materials and parts respectively, thus both can be directly implemented in the industry. The mechanical properties of ABS/ZnO nanocomposites and ABS/ZnO microcomposites 3D printed samples are presented and compared using five different filler concentrations: 0.5, 2.5, 5, 10 and 20% wt. of ZnO particles. The physical properties of the produced nanocomposites and specimens, such as their microhardness, are also addressed and presented. The main objective of this work was to manufacture nano composites and microcomposites with improved mechanical properties compared to the polymer matrix material, and at the same time evaluate the effect of the two filler scales on the mechanical properties of the polymer matrix material.

## 2. Materials and Methods

### 2.1. Materials Tested

The polymer matrix used in this work was Acrylonitrile Butadiene Styrene (ABS) procured from INEOS Styrolution (Frankfurt, Germany). The ABS used was industrial grade in fine powder form, under the name Terluran Hi-10. The nanomaterial selected as a nano filler for this work was the Sigma-Aldrich Zinc Oxide (ZnO nano, 677450, Sigma-Aldrich, St. Louis, MO, USA), with less than 50 nm particle size and assay >97%. The micromaterial selected as the micro-filler, was Sigma-Aldrich Zinc Oxide (ZnO micro, 96479) with an average particle size less than 5 μm and assay >99%.

### 2.2. Work Methods

**Figure 1:**
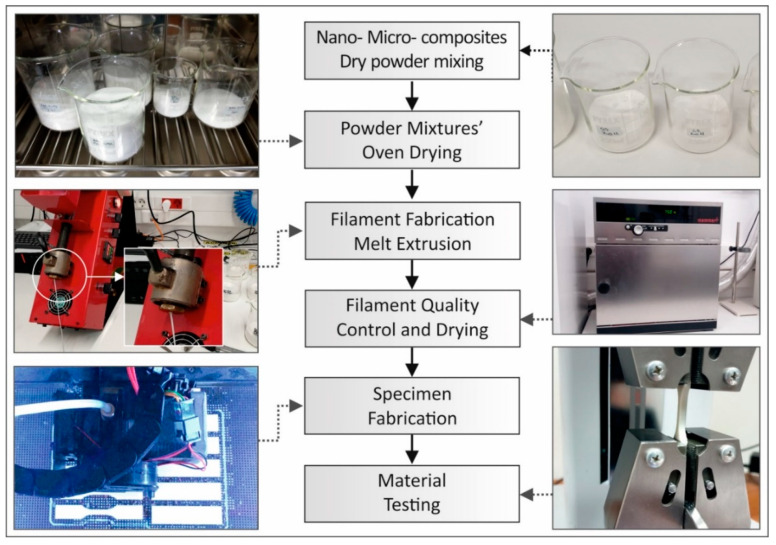
Nano- and micro-composite filaments’ and specimens’ fabrication methodology.

The ABS matrix material was physically mixed with ZnO fillers at different concentrations via a mechanical homogenizer. The result from this procedure was a homogenized powder mixture with different amounts of ZnO filler for each case studied (0.5, 2.5, 5, 10 and 20% wt.) (Figure 1).

The powder mixtures were then dried in a laboratory oven, at 70 °C for 48 h in order to achieve optimum working conditions for the extrusion system. The powder mixtures where then fed into a Noztek Pro (Noztek, Shoreham, UK) single screw extruder, preheated at 230°C. This working temperature was chosen experimentally, so that the extrusion system could produce a constant 1.75-mm diameter filament with good surface quality, while maintaining uninterrupted extrusion flow. Samples from the nanocomposite and microcomposite filaments were initially analyzed before being used for 3D printing.

The commercially available desktop 3D printer MakerBot replicator 2x (Makerbot, New York, NY, USA) was used in all cases. The specimens were built with the same 3D printing parameters, i.e., 100% solid infill, 0.2 mm layer height, 235 °C printing temperature, 110°C heat bed temperature during the 3D printing process and 45 degrees deposition orientation (Figure 1). The printing speed was set to 20mm/s and the material flow was set to 110%.

These parameters are experimentally tested to provide better properties to the specimens, since the 3D printing parameters are crucial in terms of mechanical anisotropy for the sample specimen preparation [10]. The tensile test specimens’ shape and dimensions were 3D printed to match the specification described by the American Society for Testing and Materials (ASTM) standard D638-02a [35], while the flexural test specimens were 3D printed according to the ASTM D790-10 standard [36].

#### 2.2.1. Structural Characterization

X-ray diffraction (XRD) (Rigaku Ultra high-resolution triple axis multiple reflection SmartLab X-ray Diffraction System, Japan) and Scanning Electron Microscopy (SEM) (FEI Company, Hillsborough, OR, USA) were used to analyze the structuring, the morphology and the distribution of ZnO within the polymer nanocomposites and microcomposites. The structural analysis was performed by the XRD method using a Rigaku Ultra high-resolution triple axis multiple reflection SmartLab X-ray Diffraction System in wide-angle measurements mode—WAXRD. The structural features (e.g., crystallinity and lattice constant) were investigated using a 9 kW rotating anode X-ray diffraction system that employs Cu Kα1 radiation (*λ* = 1.5406 Å). WA-XRD (Wide Angle X-Ray Diffraction) and patterns were recorded in θ/2θ scan at 5°/min from 10 to 60°. Each sample was mounted on an aluminum holder.

Detailed SEM characterization was performed using a (FE-SEM) Nova NanoSEM 630 (FEI Company, Hillsborough, OR, USA), equipped with an EDX detector (EDAX TEAM™, EDAX Inc., Mahwah, NJ, USA) and field emission microscope. All samples were characterized in high vacuum mode. In order to correctly perform the SEM characterization, samples were coated by thermal evaporation with a 5 nm Au thin film to avoid charging during the analysis.

#### 2.2.2. Thermal Properties

Thermogravimetric Analysis (TGA) (PerkinElmer, Waltham, MA, USA) was performed to obtain information about the working and destruction temperature of the ABS matrix selected for this work. The measurements were taken via a PerkinElmer Diamond Thermo Gravimetry / Differential Thermal Analyzer (TG/TDA) device with a heating cycle of room temperature (24 °C) to 600 °C with a heating step of 10 °C/min.

Differential Scanning Calorimetry (DSC) (PerkinElmer, Waltham, MA, USA) was also performed to obtain information about the effect of filler reinforcements on the glass transition temperature (*Tg*) of the composite materials. The measurements were taken via a PerkinElmer Diamond DSC with a temperature cycle of 50 °C to 350 °C with a heating step of 10 °C/min and then cooling back down to 50 °C. The heating was performed on air.

#### 2.2.3. Tensile Tests

Tensile specimens were manufactured as specified in the ASTM D638-02a standard (type V specimens with 3.2 mm thickness) [35]. Seven (7) specimens were fabricated for each case studied, to comply with the ASTM D638-02a standard, which requires at least five (5) specimens to be tested for each case. The tensile tests were performed using an Imada MX2 (Northbrook, IL, USA) tensile test apparatus employing standardized grips. The tensile test machine chuck was set at a 10 mm/min speed for testing. This apparatus tenses the specimen fixed within and effectively and accurately measures the developed displacement (deflection) in the specimen until it breaks. Force (Newton) versus displacement (mm) experimental data are logged in a file at 2000 Hz sampling rate. Experiments were conducted at room temperature (23 °C). The tensile strength and the tensile modulus of elasticity of the specimens were determined in these experiments, according to the instructions and formulas provided by the ASTM D638-02a standard.

#### 2.2.4. Flexural Tests

Flexural test specimens were 3D printed, according to the ASTM D790-10 standard (64 mm length, 12.4 mm width, and 3.2 mm thickness) [36]. Seven (7) specimens were 3D printed for each case studied, to comply with the ASTM D790-10 standard, which requires at least five (5) specimens to be tested for each case. The flexural tests were performed using an Imada MX2 (Northbrook, IL, USA) tensile test machine, with a setup compatible to the standard (three points test with 52 mm support span). The machine chuck was set at a 10 mm/min speed for testing. Force (Newton) versus displacement (mm) experimental data are logged in a file at 2000 Hz sampling rate. Experiments were conducted at room temperature (22 °C). The flexural strength and the flexural modulus of elasticity of the specimens were determined in these experiments, according to the instructions and formulas provided by the ASTM D790-10 standard.

#### 2.2.5. Micro-Hardness Tests

For defining the micro-hardness in Vickers scale, the ASTM E384-17 was utilized [37]. In accordance with the ASTM standard, a 0.2 kg force scale was used (1.962 N) and the overall indentation time was 10 s. All specimens had their surface polished and the indenter used was a typical Vickers diamond pyramid with an apex angle of 136°. All experiments were conducted in room temperature (23 °C) with the aid of an Innova Test 400—Vickers apparatus, capable of directly calculating the micro-hardness from the imprint’s mean average diagonals.

## 3. Results

### 3.1. Experimental Observations Regarding Filament and Specimens’ Fabrication

While developing the ABS/ZnO nanocomposite and microcomposite materials, filaments and specimens, several significant experimental observations were made. Filaments that were not appropriately dried led to poorly 2D printed structures and often led to clogged print heads. Another observation was that the reduction in the 3D printing speed to 20 mm/s and the increase in the material flow to 110% resulted in more consistent 3D printed structures.

In order to avoid applying 3D printing adhesive aids (like 3D printing glues), as well as to save the building specimens from warping or detaching from the heated 3D print bed, it was observed that is essential to have a fully closed 3D printing area and to maintain constant temperature in the 3D printing chamber.

### 3.2. Structural and Compositional Characterization

**Figure 2:**
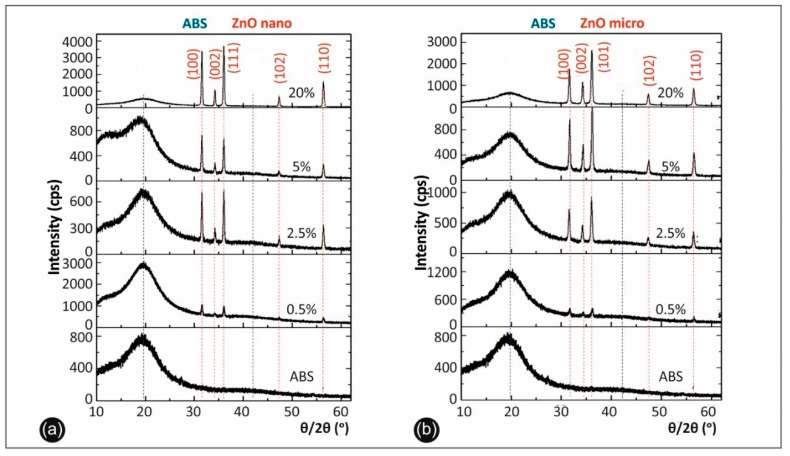
(**a**) X-ray diffraction (XRD) spectra of ABS/ZnO nanocomposites and (**b**) XRD spectra of ABS/ZnO micro-composites in concentrations studied.

The X-ray diffraction (XRD) results upon the nanocomposites and microcomposites studied are presented in Figure 2 below.

**Figure 3:**
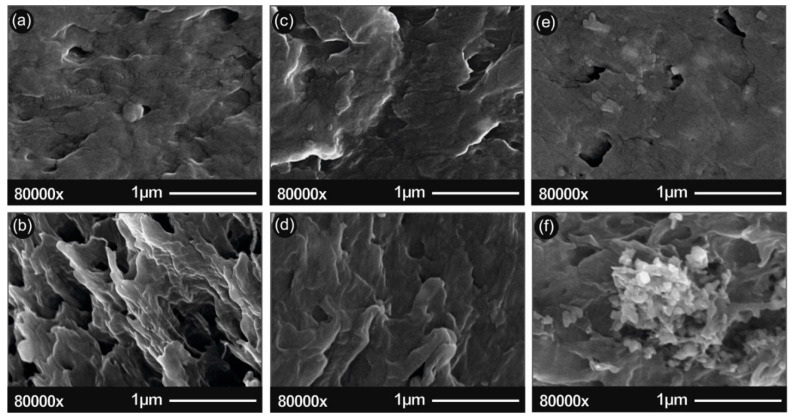
(**a**) Scanning electron microscopy (SEM) images of pure ABS surface area; (**b**) pure ABS section area; (**c**) ABS/ZnO nano 2.5% wt. surface area; (**d**) ABS/ZnO nano 2.5% wt. section area; (**e**) ABS/ZnO nano 20% wt. surface area; (**f**) ABS/ZnO nano 20% wt. section area. “Surface area” corresponds to the 3D-printed material surface, while “section area” corresponds to the surface resulted from tensile testing.

**Figure 4:**
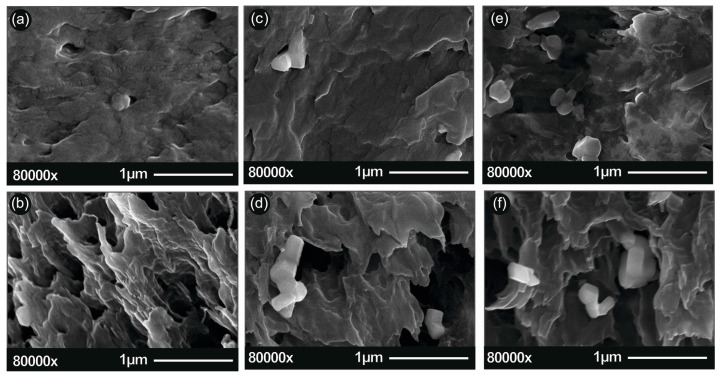
(**a**) SEM images of pure ABS surface area; (**b**) pure ABS section area; (**c**) ABS/ZnO micro 2.5% wt. surface area; (**d**) ABS/ZnO micro 2.5% wt. section area; (**e**) ABS/ZnO micro 20% wt. surface area; (**f**) ABS/ZnO micro 20% wt. section area “Surface area” corresponds to the 3D-printed material surface, while “section area” corresponds to the surface resulted from tensile testing.

In Figure 3, the SEM images of the pure ABS surface area (a), the pure ABS section area (b), the ABS/ZnO nano 2.5% wt. surface area (c), the ABS/ZnO nano 2.5% wt. section area (d), the ABS/ZnO nano 20% wt. surface area (e) and the ABS/ZnO nano 20% wt. section area (f) are presented. In Figure 4, the ABS/ZnO micro 2.5% wt. surface area (c), the ABS/ZnO micro 2.5% wt. section area (d), the ABS/ZnO micro 20% wt. surface area (e) and the ABS/ZnO micro 20% wt. section area (f) are presented. “Surface area” corresponds to the 3D-printed material surface, while “section area” corresponds to the surface resulted from tensile testing.

### 3.3. Thermal Analysis

**Figure 5:**
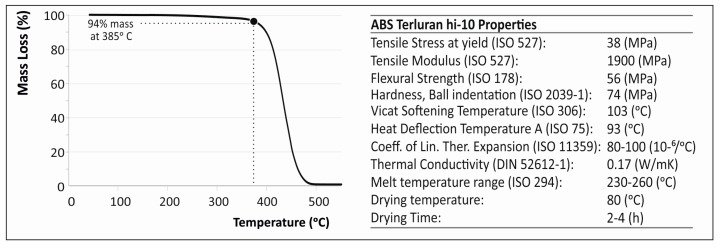
Thermogravimetric analysis (TGA) mass loss versus temperature curve for the ABS Terluran Hi-10 experimentally determined in this work and ABS polymer matrix manufacturer properties (courtesy of Ineos Styrolousion, material datasheet).

The TGA mass loss versus temperature curve for the selected ABS polymer is presented in Figure 5 below.

**Figure 6:**
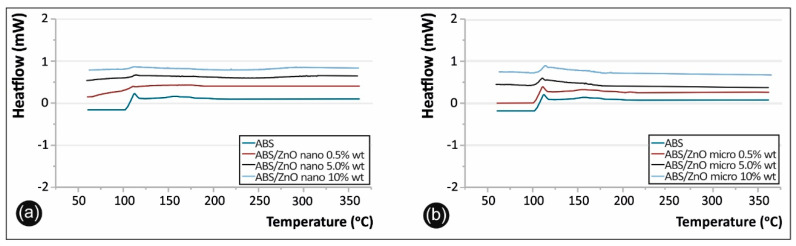
(**a**) Differential scanning calorimetry (DSC) curves for ABS, ABS/ZnO nanocomposites and (**b**) ABS/ZnO micro-composites.

The DSC heat flow versus temperature curves for the selected ABS polymer and the fabricated nanocomposites and microcomposites are presented in Figure 6 below. DSC results also showed the relaxation peaks, summarized in Table 1 below. The glass transition temperatures (*T_g_*) of the various compositions studied and the equivalent results are also summarized in Table 1.

### 3.4. Tensile Properties

**Figure 7:**
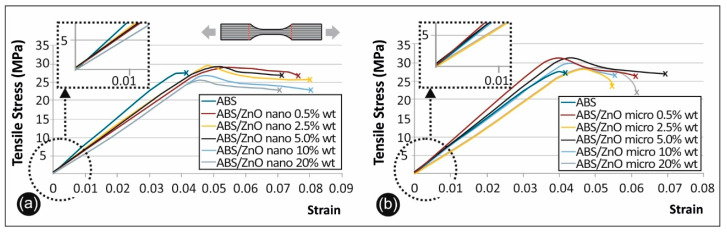
(**a**) Tensile stress vs. strain graphs for ABS/ZnO nano and (**b**) ABS/ZnO micro.

Figure 7 shows characteristic stress–strain curves derived and calculated from the tensile testing of ABS/ZnO nano (Figure 7a) and ABS/ZnO micro (Figure 7b) composites.

**Figure 8:**
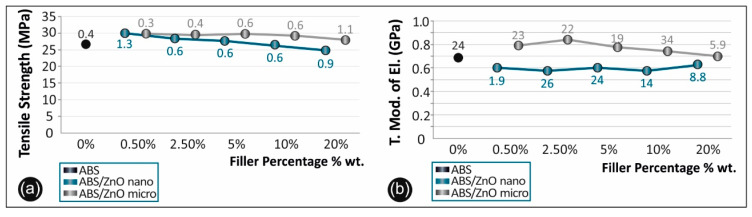
(**a**) Comparative tensile strength graph and (**b**) tensile mod. of elasticity for all the materials studied (numbers in the graph points indicate the calculated deviation for each value).

Figure 8a summarizes and compares the tensile strength of the unfilled ABS versus the ABS/ZnO nanocomposites and the ABS/ZnO microcomposites at all the concentrations studied, while Figure 8b, summarizes and compares the tensile modulus of elasticity of the unfilled ABS versus the ABS/ZnO nanocomposites and the ABS/ZnO microcomposites at all the concentrations studied.

The overall results regarding the tensile strength and the tensile modulus of elasticity of each case studied of ABS, ABS/ZnO nano and ABS/ZnO micro are presented in Table 2 below. Table 2 also presents, for each composite, the average values of the calculated mechanical properties along with their deviations, in all cases studied.

### 3.5. Flexural Properties

**Figure 9:**
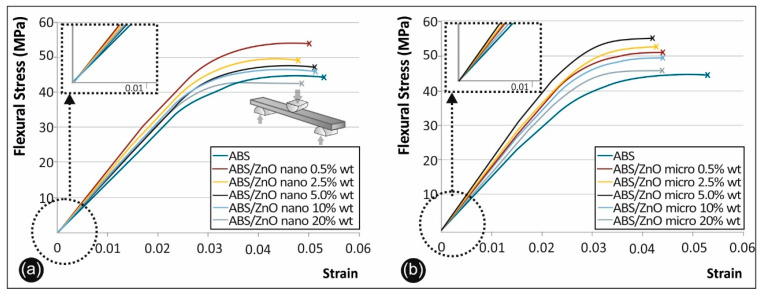
(**a**) Flexure stress vs. strain graphs for (**b**) ABS/ZnO nano and ABS/ZnO micro.

Figure 9 shows characteristic stress–strain curves derived and calculated from the flexural testing of ABS/ZnO nano (Figure 9a) and ABS/ZnO micro (Figure 9b) in respect to pure ABS.

**Figure 10:**
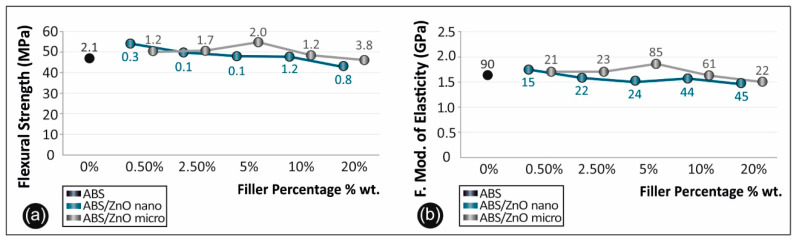
(**a**) Comparative flexural strength graph and (**b**) flexural mod. of elasticity for all the materials studied (numbers in the graph points indicate the calculated deviation for each value).

Figure 10a summarizes and compares the flexural strength of the unfilled ABS versus the ABS/ZnO nanocomposites and the ABS/ZnO microcomposites at all the concentrations studied, while Figure 10b summarizes and compares the flexural modulus of elasticity of the unfilled ABS versus the ABS/ZnO nanocomposites and the ABS/ZnO microcomposites at all the concentrations studied.

The overall mechanical properties results’ regarding the flexural strength and the flexural modulus of elasticity of each case studied of ABS, ABS/ZnO nano and ABS/ZnO micro are presented in the Table 2 below:

### 3.6. Micro-Hardness Results

**Figure 11:**
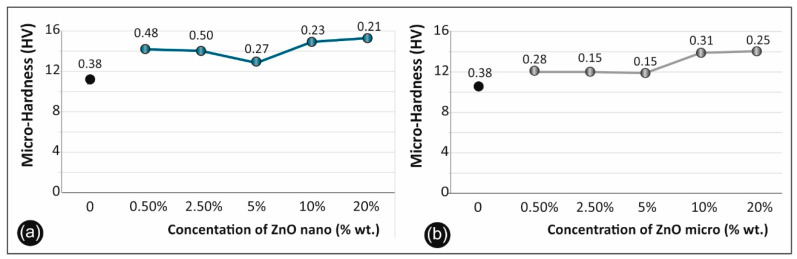
Micro-Hardness Vickers results of ABS/ZnO nanocomposites (**a**) and micro-composites (**b**) versus the filler concentration (numbers in the graph points indicate the calculated deviation for each value).

Regarding the micro-hardness testing of the materials studied, the results are summarized in Figure 11a for the cases of ABS/ZnO nano and in Figure 11b for the cases of ABS/ZnO micro.

## 4. Discussion

### 4.1. Structural and Compositional Characterization

XRD characterization of ABS/ZnO nanocomposite and microcomposite materials was performed in order to study the nano-/micro-materials concentration effects on the composite structure (Figure 2). The peak indexing for ZnO was made according to the ICDD database with card number 01-080-0075. The presence of ZnO was identified with the hexagonal spatial group 186:P63mc with a = b = 0.32 nm and c = 0.52 nm. It can be observed that the ZnO nanoparticles exhibit broader diffraction peaks compared to ZnO nanoparticles. This behavior can be attributed to lower crystallite sizes in the case of ZnO nanoparticles, according to the Debye–Scherrer formula. This gives the correlation between the Full Width at Half Maximum (FWHM) (*β*) by means of the crystallite size (*d*):

*d* = *kλ*/(*β*cosθ) (1)

where *k* is a shape factor taken equal as 0.9, 2ϴ is the peak angular position and λ is the wavelength. Taking into account the (101) reflection for ZnO nanoparticles, it was obtained that the mean crystallite size varies between 27.7 and 34.1 nm, while for ZnO microparticles the mean crystallite size reaches up to 36.6 nm, when increasing the ZnO concentration. The results strongly suggest an increase in the crystallinity with the increasing ZnO concentration.

In order to study the influence of ZnO concentration in the ZnO crystallinity, the three most intense diffraction peaks were investigated: (100), (002) and (101), respectively. After carefully investigating the FWHM of the peaks, it can be observed that the FWHM of the diffraction peaks has a dependence on the ZnO concentration for both nanoparticles and microparticles for all reflections. To minimize the other effects in the Bragg peaks, the instrumental broadening, ${\left(\beta_{hkl}\right)}_{instrumental}$, which is around 0.03° in the parallel-beam mode, was subtracted from the width of the diffraction peaks, ${\left(\beta_{hkl}\right)}_{measured}$ in the following way:

(2) $$\beta_{hkl}={\left[{\left(\beta_{hkl}\right)}_{measured}^{2}-{\left(\beta_{hkl}\right)}_{instrumental}^{2}\right]}^{1/2}$$

As shown in Table 3; Table 4, FWHM decreases when the ZnO concentration is increased, which is ascribed to the increase in the mean crystallite size. The mean crystallite size was recalculated using the Scherrer formula, considering the most intense diffraction peak, namely (101). Accordingly, in the case of nanoparticles, the mean crystallite size increases from 27.7 nm (0.5%) to 28.7 nm (2.5%) to 31.5 nm (5%), reaching to 34.1 nm at 20%. On the other hand, in the case of microparticles, the mean crystallite size increases from 35.27 nm (0.5%) to 36.1 nm (2.5%), 36.3 nm (5%), till 36.6 nm (20%).

At the same time, the ratio between intensities of the diffraction peak corresponding to ABS and ZnO nanoparticles and microparticles decreases when the micro/nanomaterials concentration increases. The peak intensity variations can be related to mass variation as well as to structural changes. These observations support the idea that the micro/nanoparticles were successfully embedded in polymeric matrix.

High resolution SEM characterization was used to analyze the nano/micro structuring of both the surface of the 3D printed material as well as the section of the 3D printing specimens. Figure 3 and Figure 4 present typical images of the 3D-printed material (surface and section) for pure ABS and nano composites and microcomposites with 2.5% and 20% of ZnO nano/micro filler, respectively.

As can be seen in the SEM images, ZnO (white granular grains with geometric shapes in the images) embeds uniformly in the polymeric matrix. A higher ZnO nano concentration seems to promote nanomaterials agglomerations. One such example is shown in Figure 3f.

Figures denoted “section”, present the fracture areas of the tensile specimens of pure ABS and nanocomposites and microcomposites with 2.5% and 20% of ZnO nano/micro filler, respectively. As it can be seen, a low ZnO nano concentration leads to elastic fracture and thus larger breaking “spikes” on the fracture surface when compared to pure ABS. In contrast, higher concentrations seem to promote a more brittle fracture behavior with a ”cleaner” breaking section. This is because the ZnO filler presence in the ABS matrix dramatically changes the mechanical properties of ABS, as it is presented in the following sections.

### 4.2. Thermal Analysis

From the TGA analysis, it was shown that the working temperature of the selected ABS must be below the critical temperature of 384 °C, were the ABS starts degrading and rapidly losing mass. At this point, it must be mentioned that ABS is amorphous and therefore has no true melting point. Furthermore, ABS has a reported *T_g_* value of approximately 105 °C.

From the DSC analysis, it was shown that for almost every case studied, *T_g_* temperature had no substantial variations, leading to the assumption that the specimens have homogeneous distribution of the filler. For the case of ABS/ZnO micro with 10% wt., a slight increase in *T_g_* compared to the values of the composites with a lower filler percentage was found, indicating that the hindering effect on the ZnO, responsible for the decrease in the mobility of the ABS chains, occurred noticeably at higher filler contents.

As stated before, nanoscale interactions can significantly alter the mechanical and physical response of a selected material. The current study and reports in literature have shown that filler particles agglomerations within the polymer matrix are a cause for variations in Glass Transition Temperature (*T_g_*) and also contribute to the concentration of loading stresses, reducing the overall mechanical properties of the final composites [4,38].

### 4.3. Tensile Test Results

Comparing the ABS/ZnO nano and the ABS/ZnO micro materials it was evident that the ABS microcomposites developed higher mechanical strength than those of ABS/ZnO nano materials. This percentage is approximately 3% when all concentration cases are taken into account. There is also an increase in the tensile modulus of elasticity. The overall average increase in the tensile modulus of elasticity is about 7%, demonstrating a stiffer behavior.

On the other hand, as the nanoparticles agglomerate, there are several possible mechanisms that might lead to an increase in the material strength—one being their interaction with the polymer matrix [39]. The effective size of the filler has an important role in determining the mechanical properties of the final composite. There is a general rule that as the size of the filler particles decrease, the effective filler surface area increases along with the interactions with the matrix.

On the other hand, it was shown that when the surface area to volume ratio decreases, the total number of particle–polymer interactions also decrease. Studies have proven that if there is strong interfacial bonding between the filler and the matrix, because of the high specific surface area of the filler particles, this can enhance the overall mechanical performance of the matrix when compared to the micro-composite [14,15,16,17,18,40,41].

On the contrary, there could be a possible scenario that in the case of micro fillers, with smaller surface area than that of nano-scaled fillers, an increase in the mechanical strength can be expected, due to an adverse effect on the strength of the polymer matrix leading to decreased number of interactions [4,39]. This scenario is consistent with other literature reviews that state that there is increased polymer flexibility near the nanoparticles that leads to a decrease in *T_g_* [38,39].

Moreover, it was shown that at a higher filler loading, the polymer chains become immobilized, while there is a significant concentration of stress upon the points of agglomeration. This induces fracture points [35] and thus lowers the mechanical properties of the composite. The increase found in the current research in the tensile strength and in the tensile modulus of elasticity of ABS/ZnO microcomposites when compared to ABS/ZnO nanocomposites confirms the above scenario.

### 4.4. Flexural Test Results

For the case of ABS/ZnO nano, it was found that the optimum filler loading is, again, 0.5% wt. Zinc Oxide, leading to a 15% increase in the flexural strength when compared to unfilled, pure ABS. For the case of the ABS/ZnO micro it was shown that the optimum filler loading is 5% wt. Zinc Oxide, leading to a 17% increase in the flexural strength when compared to unfilled, pure ABS. From the flexural stress–strain curves it was observed that all the nanocomposite specimens, depending on the filler’s concentration, had more brittle fracture when compared to pristine ABS specimens. This was also evident in the fracture area of the filaments and the fracture areas of the 3D-printed nanocomposite specimens under examination.

Comparing the ABS/ZnO nano and the ABS/ZnO micro materials, it was evident that the ABS micro-composites developed approximately 1.3% higher flexural strength than those of ABS/ZnO nano materials when all concentration cases were considered. There was also an increase in the flexural modulus of elasticity. The overall average increase in the flexural modulus of elasticity is about 3%. These findings are similar to the tensile strength results reported above, while they are less pronounced, confirming that there is a pattern.

### 4.5. Micro-Hardness Results

Regarding the micro-hardness results, depicted in Figure 11, it was evident that the ABS/ZnO nanocomposites (Figure 11a) had higher micro-hardness values when compared to pure ABS and to ABS/ZnO micro-composites (Figure 11b). Both composites studied exhibited the same trend; at 0.5% wt. concentration, both showed increased micro-hardness when compared to pure ABS. Both composites’ hardness kept reducing until 5% wt. filler concentration, while after 5% wt., the micro-hardness kept rapidly increasing, reaching the peak value at 20% wt. filler concentration. The increase in the microhardness, especially after the 10% wt. concentration, is due to structuring alterations that the fillers induced in the ABS matrix.

## 5. Conclusions

**Figure 12:**
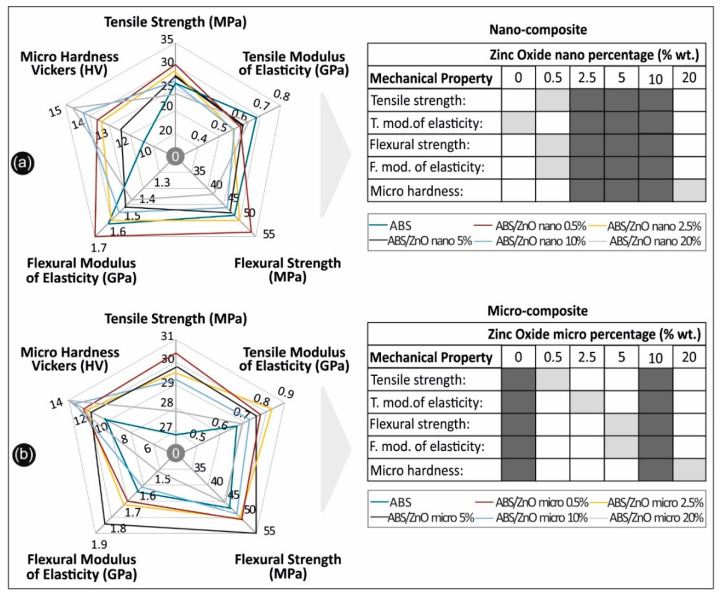
Overall comparative graphs for (**a**) ABS/ZnO nanocomposites and (**b**) ABS/ZnO micro-composites, in all scenarios studied.

In this study nanocomposite and microcomposite filaments comprising of ABS and Zinc oxide were developed in various concentrations, for application in Fused filament fabrication (FFF). It was proven that with the fabrication methodology described herein, it is possible to fabricate physically and mechanically improved, homogeneous nanocomposite or microcomposite materials for direct commercial or industrial implementation, while incorporating a viable, clean (solvent-free), commercially applicable methodology. From the overall assessment of the results, which are summarized in Figure 12, it can be concluded that the implementation of nano and micro fillers can not only alter the mechanical and physical response of a polymer matrix, but also noticeably improve it. The micro fillers in this study had a slightly higher overall impact on the increase in the polymer mechanical properties than the nano fillers. It was also evident that there is a percolation threshold—a specific filler concentration value—that provides the optimum mechanical properties for each material studied. For the ABS/ZnO nanocomposites, this value was proven to be 0.5% wt., while for the case of the ABS/ZnO micro-composites, this value was 0.5% wt. for the tensile strength and 5% wt. for the flexural strength.

## Figures and Tables

**Table 1:** *T_g_* and relaxation temperatures for materials studied.

Material	*T_g_* (°C)	Relaxation (°C)
ABS	107	112
0.5% wt. ZnO nano	107	112
5.0% wt. ZnO nano	107	111
10.0% wt. ZnO nano	108	111
0.5% wt. ZnO micro	107	112
5.0% wt. ZnO micro	107	111
10.0% wt. ZnO micro	110	113

**Table 2:** Tensile and flexural properties and deviations of each material studied.

Material	Tensile Strength (MPa)	Youngs Modulus (GPa) *	Flexural Strength (MPa)	Flexural Modulus (GPa) *
ABS	26.7 ± 0.4	0.69 ± 0.02	46.8 ± 2.2	1.64 ± 0.09
0.5% wt. ZnO nano	30.3 ± 1.3	0.60 ± 0.00	54.0 ± 0.3	1.76 ± 0.00
2.5% wt. ZnO nano	38.3 ± 0.6	0.58 ± 0.00	49.9 ± 0.2	1.61 ± 0.02
5.0% wt. ZnO nano	27.8 ± 0.6	0.60 ± 0.02	48.0 ± 0.1	1.52 ± 0.02
10.0% wt. ZnO nano	26.6 ± 0.6	0.58 ± 0.01	47.3 ± 1.2	1.57 ± 0.04
20.0% wt. ZnO nano	24.8 ± 0.9	0.63 ± 0.00	43.2 ± 0.9	1.48 ± 0.04
0.5% wt. ZnO micro	30.4 ± 0.3	0.79 ± 0.02	50.5 ± 1.2	1.71 ± 0.02
2.5% wt. ZnO micro	29.6 ± 0.4	0.84 ± 0.02	50.5 ± 1.7	1.71 ± 0.02
5.0% wt. ZnO micro	29.8 ± 0.6	0.78 ± 0.01	54.8 ± 2.0	1.86 ± 0.08
10.0% wt. ZnO micro	29.2 ± 0.6	0.74 ± 0.03	48.4 ± 1.2	1.63 ± 0.06
20.0% wt. ZnO micro	27.9 ± 1.1	0.70 ± 0.00	46.1 ± 3.8	1.52 ± 0.02

* Zero deviation in these columns implies that the deviation requires more than two decimal digits to be presented.

**Table 3:** The FWHM of the diffraction peaks on (100), (002) and (101) at 0.5%, 2.5%, 5% and 20% concentration of ZnO nanoparticles.

Reflection (hkl)	$\beta_{hkl}({}^{{}^{\circ}})—0.5\%$	$\beta_{hkl}$ (°)—2.5%	$\beta_{hkl}\;({}^{{}^{\circ}})—5\%$	$\beta_{hkl}\;({}^{{}^{\circ}})—20\%$
(100)	0.341	0.331	0.292	0.269
(002)	0.321	0.303	0.285	0.271
(101)	0.334	0.323	0.294	0.272

**Table 4:** The FWHM of the diffraction peaks on (100), (002) and (101) at 0.5%, 2.5%, 5% and 20% concentration of ZnO microparticles.

Reflection (hkl)	$\beta_{hkl}\;({}^{{}^{\circ}})—0.5\%$	$\beta_{hkl}\;({}^{{}^{\circ}})—2.5\%$	$\beta_{hkl}({}^{{}^{\circ}})—5\%$	$\;({}^{{}^{\circ}})—20\%$
(100)	0.260	0.262	0.254	0.252
(002)	0.258	0.254	0.249	0.245
(101)	0.263	0.257	0.255	0.253
